# Evaluation of effectiveness of photobiostimulation in alleviating side effects after dental implant surgery. A randomized clinical trial

**DOI:** 10.4317/medoral.23336

**Published:** 2020-01-22

**Authors:** Gianluigi Caccianiga, Letizia Perillo, Marco Portelli, Marco Baldoni, Cosimo Galletti, Cosme Gay-Escoda

**Affiliations:** 1 Assistant Professor, School of Medicine and Surgery, University of Milano-Bicocca, Monza, Italy; 2 Full Professor, Multidisciplinary Department of Medical-Surgical and Dental Specialties, University of Campania "Luigi Vanvitelli", Napoli, Italy; 3 Assistant Professor, Department of Biomedical and Dental Sciences and Morphofunctional Imaging, School of Dentistry, University of Messina, Italy; 4 Full Professor, School of Medicine and Surgery, University of Milano-Bicocca, Monza, Italy; 5 DDM, MS. Professor of Integrated Adult Dentistry. School of Dentistry. University of Barcelona. Researcher of the IDIBELL Institute; 6 MD, DDS, PhD, MS, EBOS, OMFS. Charmain and Professor of the Oral and Maxillofacial Surgery Department. School of Dentistry, University of Barcelona. Coordinator/Researcher of the IDIBELL Institute. Head of Oral and Maxillofacial Surgery, Department of the Teknon Medical Center, Barcelona, Spain

## Abstract

**Background:**

To assess if photobiostimulation (PBS) alleviates pain intensity/duration and swelling after implant surgery.

**Material and Methods:**

Sixty subjects (27 male and 33 female, with a mean age of 47,13 8.05 years) were included and randomly assigned to experimental group (implant surgery and photobiostimulation), placebo group (implant surgery and simulated photobiostimulation) and control group (implant surgery only). Inclusion criteria: subjects older than 20 years, with a healthy oral mucosa and requiring implant surgery. Exclusion criteria: pregnancy, history of implant failure, light sensitivity, metabolic deseases, consumption of antibiotics or corticosteroids in the last two weeks, smokers and alcohol drinkers. Patients reported the pain experienced by using a numeric rating scale (NRS) at 2 hours, 6 hours, 12 hours, 24 hours and from day 2 to 7. Swelling score was assessed by linear measurements and type and number of analgesic drugs within each time-point were recorded on a spreadsheet. Data of pain and amount of swelling were compared among the three groups by using the Kruskal-Wallis H Test and post-hoc comparisons tests.

**Results:**

Pain in the experimental group was less compared to controls and placebo group, at each time intervals (*p* < 0.001) as well as the maximum pain score (experimental group: median = 2, interquartile range 2-3; control group: median = 8, interquartile range 3,75-9; placebo group: median = 8, interquartile range 6,25-9). Swelling was almost insignificant in the experimental group (maximum value = 1, interquartile range 0-2,75, at 24 hours) compared with control (maximum value = 6, interquartile range 5-8,75, at 24 hours) and placebo (maximum value = 6, interquartile range 5-8, at 24 hours). Subjects in the experimental group assumed less analgesics compared to both controls and placebo groups.

**Conclusions:**

Photobiostimulation is an effective method to reduce pain intensity/duration and swelling after implant surgery.

** Key words:**LLLT, photobiostimulation, pain, implant surgery.

## Introduction

Dental implant therapy has revolutionized the treatment of all forms of edentulism (1). However, specific side effects have been reported after surgical procedure for dental implant placement such as bleeding, pain, swelling, paresthesia, nerve damage, infection and implant failure (2-4). Preventing or reducing the risk of inflammation after implant surgery become fundamental in order to reduce pain, swelling, and infection. In this respect, clinicians use similar drugs for different treatments and there is no general consensus to prescribe the drugs based on the patient’s health status (5,6).

Photobiostimulation is a side effects-free therapy with several applications in medicine and modern dentistry (7-9). It uses low-powered light within the red to near-infrared range (wavelengths from 632 to 1064 nm) to induce biological reaction. Previous studies demonstrated that photobiostimulation can effectively accelerate healing and pain relief through reduction of mediators and inflammatory cells and increased endorphin, respectively (10). Moreover, it seems to reduce orthodontic pain on two levels: 1) by inhibiting the release of arachidonic acid, which reduces the levels of prostaglandin E2 (11,12) and 2) by provoking the release of beta-endorphin which induces an effective analgesic reaction (13,14). In this respect, a recent well-conduceted trials confirmed that photobiostimulation can be used to reduce the severity and duration of pain after dental implant surgery (15). Also, it can reduce facial swelling and accelerate wound healing (15). However, more high quality randomized clinical trials (RCTs) are needed confirm or reject these findings.

In this respect, the aim of the present randomized clinical trials was to assess the effectiveness of photobiostimulation in controlling and/or reducing pain and swelling after dental implant surgery by using the spectral technology-based ATP38® device (Biotech Dental, Allée de Craponne, Salon de Provence, France).

## Material and Methods

This randomized, with 3 parallel groups (1:1:1), single operator, clinical trial was approved by the ethics committee of the Faculty of Medicine at the Milano-Bicocca University (protocol n. 11/17) and was conducted in observance of the Declaration of Helsinki. Subjects were selected and treated between March 2017 and April 2019 and all patients signed an appropriate informed consent.

- Human subjects

Sixty patients were selected from a larger pool of subjects [283] who needed implant surgery in a private dental clinic in Bergamo (Italy). Study power was investigated taking into account data obtained by a recent study (14) demonstrating a difference of 2 points in the pain experienced 24 hours after implant surgery between photobiostimulation and control group, measured via visual analogue scale (VAS). A sample size of 54 participants (18 for each groups) was considered sufficient to obtain 80% power at a 95% confidence interval, however, to balance for potential incompleteness of data, we decided to include 20 subjects in each group (tested, control and placebo).

Patients were enrolled based on the following criteria: subjects older than 20 years, with a healthy oral mucosa and requiring implant surgery. Exclusion criteria: pregnancy history of implant failure, light sensitivity, metabolic deseases, assumption of antibiotics or corticosteroids in the last two weeks, smokers and alcohol drinkers. A randomized balanced block protocol using sex and age as stratification factors was performed to randomly allocate subjects to receive implant surgery plus administration of LLLT (experimental group, 20 subjects), implant surgery only (control group, 20 subjects) and implant surgery with simulated administration of LLLT (placebo group, 20 subjects). For randomization purposes, SPSS Statistics software (IBM Corporation, Armonk, USA) was used to generate an allocation sequence.

Despite some patients reported multiple edentulia, they were enrolled in this study to underwent a single dental implant surgery. Each dental implant was inserted by Cone-beam computed tomography (CBCT) guided surgery (iCAT CBCT Unit, Imaging Sciences International, Hartfield, PA, USA), in particular a 3d printed surgical guide (Formlabs SLA 3D printer, Somerville, MA, USA) was created for each patient in order to standardized the surgical procedure. Data score of pain and swelling were recorded only after the first single implant surgery in those patients.

- Intervention

**Figure 1:**
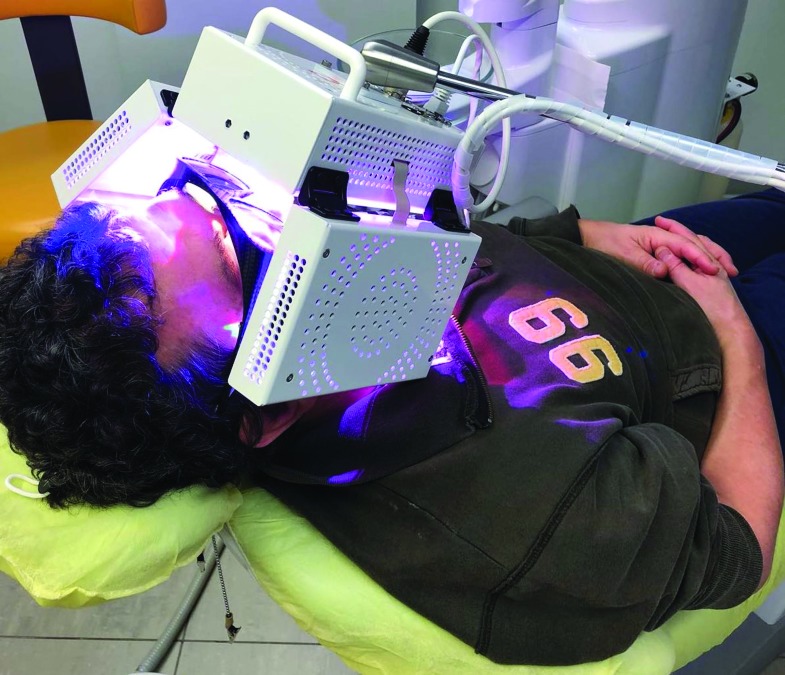
The ATP38® (Biotech Dental, Allée de Craponne, Salon de Provence, France) used in this study to perform photobiomodulation.

After implant surgery was performed, patients in the experimental group received Photobiostimulation by using the ATP38® (Biotech Dental, Allée de Craponne, Salon de Provence, France). This device features a multi-panels system emitting cold polychromatic lights with a combination of wavelengths from 450 to 835 nm depending on the field of action, i.e., the part treated and the therapeutic indication (healing, anti-inflammatory, and analgesic effect) (Fig. 1).

For the purpose of the present investigation, the analgesic module was selected according to the manufacturer instructions. Irradiation parameters were different for each type of cold light source (total duration = 730 seconds): blue light (wavelength 470 nm, duration 676 seconds out of total 730 seconds, fluency 9 joule/cm2, frequency 70 hertz), green light (wavelength 525 nm, duration 544 seconds out of total 730 seconds, fluency 3 joule/cm2, frequency 70 hertz), red light (wavelength 620 nm, duration 730 seconds out of total 730 seconds, fluency 6 joule/cm2, frequency 70 hertz), deep red light (wavelength 680-760 nm, duration 721 seconds out of total 730 seconds, fluency 18 joule/cm2, frequency 70 hertz) and infrared light (wavelength 800-835 nm, duration 655 seconds out of total 730 seconds, fluency 12 joule/cm2, frequency 70 hertz). The Photobiostimulation procedure was entrusted to a single operator (G.C.).

All patients were recommended to take Azithromycin 500 mg (Pfizer. Latina, Italy) once daily for three days i.e., from the day before to the day after implant surgery and to control chemical plaque by using chlorhexidine mouthwash (0.12%) for a period of two weeks. Analgesics were not prescribed but could be used in case of acute pain, however patients must have reported the type and number of analgesic drugs taken within each time-point (see below).

- Assessment of pain, swelling and possible drugs (analgesic) assumption

All subjects were instructed to describe the intensity of pain experienced by using a numeric rating scale (NRS), raging from 0 to 10, at specific time-points i.e., 2 hours, 6 hours, 12 hours, 24 hours and from day 2 to 7 (14,16). The assessment of facial swelling was performed by using linear measurements (15) at 24 hours and from day 2 to 7 after surgery. Moreover, type and number of analgesic drugs within each time-point were recorded on a spreadsheet. The procedure of data collection and assessment of facial swelling was entrusted to another operator who was blinded about the three groups of the study (A.L.)

- Statistical analysis

The Shapiro-Wilk test was used to investigate the normal distribution of the data set. As data of pain and swelling were not normally distributed, inferential statistics was performed for these two outcomes using non-parametric tests. Kruskal-Wallis H Test was used to assess if there were differences 1) in the maximum pain and in the pain experienced at each time interval among experimental, control and placebo groups and 2) in the amount of swelling among the three groups. Post-hoc comparisons were performed using the Dunn's multiple comparison test. As data from drug assumptions were normally distributed, the one-way analysis of variance (ANOVA) was performed to verify any statistical significance in the assumption of analgesic among the three groups investigated with Bonferroni’s test used for assessing multiple comparisons between groups.

## Results

**Figure 2:**
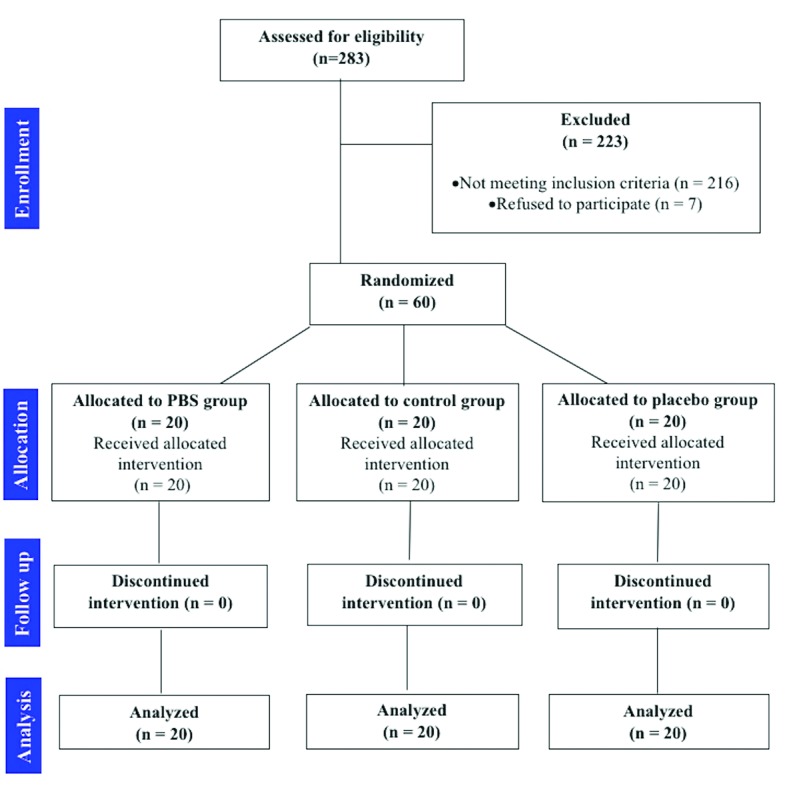
CONSORT flowchart.

In the present randomized clinical trial we did not face with drop-out, thus the final sample consisted of 60 patients, 27 male and 33 female, with a mean age of 47.13 8.05 years. The demographic and clinical characteristics of the sample of the study are shown in Table 1, also the CONSORT flowchart is reported in Fig. 2.

**Table 1:**
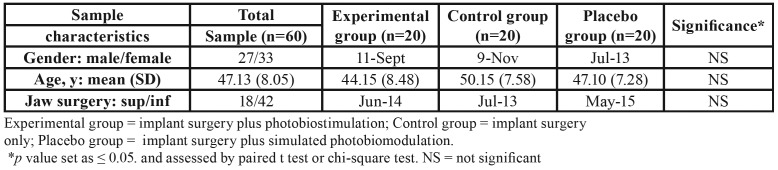
Demography, clinical characteristics and descriptive statistics of the study sample.

**Figure 3:**
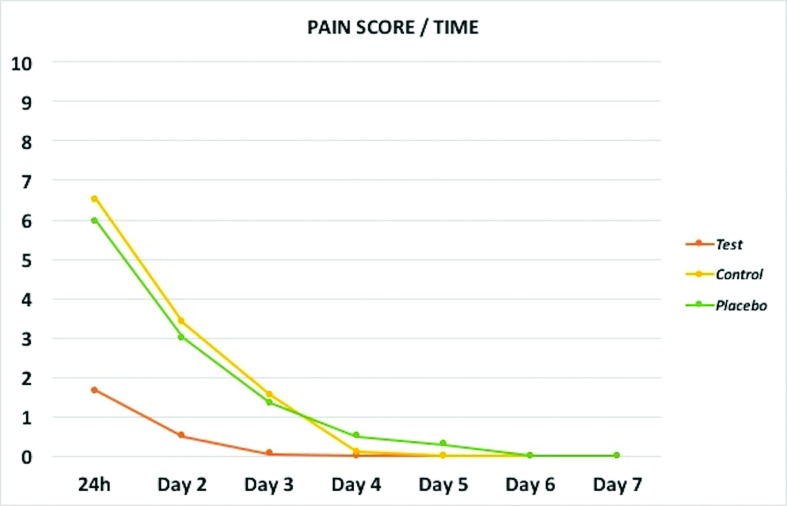
Graph showing levels of pain reported in each group of subjects at each time point.

Fig. 3 shows the levels of pain reported in each group at each time point. In particular, in the experimental group pain experienced is less and decrease even before compared to subjects in both control and placebo groups as confirmed by the Kruskal-Wallis H test and the Dunn's multiple comparison test (Table 2). In this respect, the maximum pain experienced in the experimental group was, in median value, 2 point on the NRS while it reached 8 point in both control and placebo group.

**Table 2:**
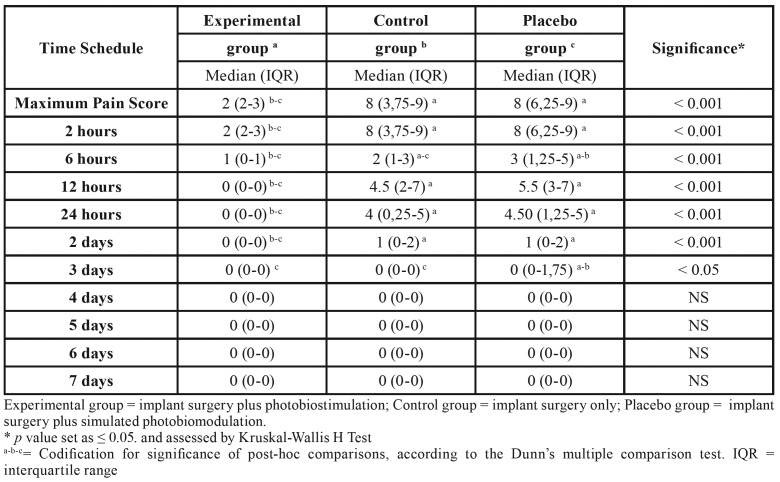
Maximum pain score and pain experienced at each time schedule, assessed via Numeric Rating Scale (NRS).

The level of swelling reported by patients was found to statistically differ among the three groups (Table 3), according to the Kruskal-Wallis H test. In this respect, swelling was almost insignificant in the experimental group were the maximum median value was 1 at 24 hours after surgery; on the contrary, both control and placebo group reported a median value of swelling of 6 at 24 hours which gradually decrease up to day 4, i.e. when the median values reported was 0. Thus, no differences were found between control and placebo groups, as assessed by the Dunn's multiple comparison test.

Finally, the assumption of drugs differs among the three groups, according to the ANOVA on-way analysis of variance (Table 4).

**Table 3:**
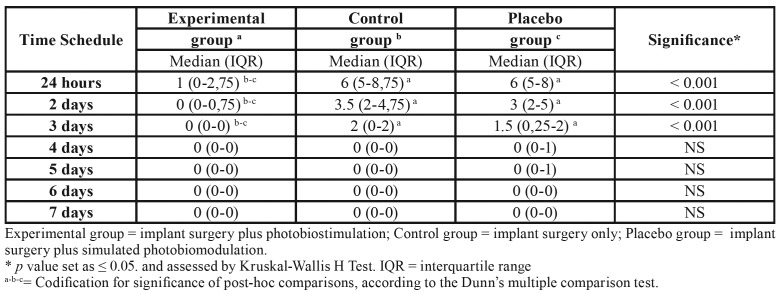
Median score of swelling from 24 hours to 7 days after implant surgery.

**Table 4:**
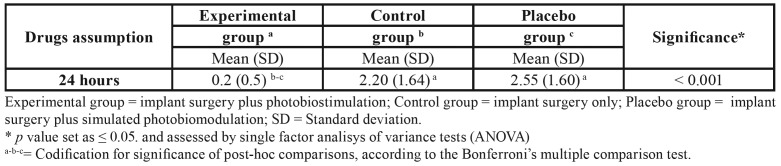
Mean values of drugs assumption after implant surgery among each groups investigated.

In particular, subjects in the experimental group reported a total mean value of analgesics (pills) of 0.2, while the control and placebo groups reported respectively a mean value of 2.20 and 2.55. Thus, no differences were found between control and placebo groups, as assessed by the Bonferroni multiple comparison tests.

## Discussion

Edema and swelling are the most frequent side effects reported after dental implant surgery. As consequence, the management of these side effects becomes crucial in order to enhance the patients’ post-surgical experience and increase their comfort (17).

The beneficial rule of photobiostimulation in reducing pain after surgical procedure is still controversial with some studies reporting no significant advantages (18,19) and other studies reporting a significant effectiveness of photobiostimulation in reducing pain compared to control group (15,17). In this respect, Safdari *et al*. (15) reported a significant reduction in pain duration and also pain intensity at 12, 24, 48, 72 hours after implant surgery in the laser group compared with control group.

In the present study, we found a significant reduction in the pain experienced in the experimental group (implant surgery plus photobiomodulation) compared to both control (implant surgery only) and placebo (implant surgery and simulated photobiomodulation) in each time-point up to day 3 (Table 2). According to Farrar *et al*. (20), the benchmark of clinical significant change in the experience of pain corresponds to a reduction/augmentation of approximately two points in the NRS. In this respect, the differences in maximum pain score and in the pain recorded at specific time points (2 hours, 12 hours and 24 hours 3) between experimental and control group were equal or higher than two points on the NRS scale, corroborating the clinical significance of our findings. As showed in Fig. 3, we also found a reduction of pain duration after photobiostimulation since pain reported in this group completely disappeared 24 hours after surgery (all patients in the tested group reported 0 value from this time-point on). As consequence of the reduction of pain intensity and duration, we found that subjects in the experimental group turned to analgesic drugs significantly less than subjects in the control/placebo groups for the management of post-surgical pain.

Moreover, subjects in the experimental group presented a significant reduction in the score of facial swelling compared to both control and placebo groups, at least up to day 3 since, from this time-point after, swelling almost disappear even in the latter groups. These findings would corroborate the use of photobiostimulation to control post-surgical swelling and could be attributed to its rule in facilitating vasodilatation, increasing circulation, phagocytosis and ymphatic drainage (15,21-23).

One of the interesting aspects of the present study was the use of ATP38® to perform photobiostimulation. This device features a multi-panels system emitting cold polychromatic lights with a simultaneous combination of wavelengths that correspond to Cytochrome C Oxidase and Porphyrin absorption peaks (from 450 to 835 nm); this correspondence, similar to 7 windows open over different depths, favours the power density, i.e., a strong concentration of photons to quickly deliver the total intended energy dose. This is called spectral technology. In the light of the present findings, the ATP38 can be considered a valuable tool for the management of post-operative implant surgery side-effects. Further prospective clinical trials must be carried out to assess if spectral technology is more effective than conventional single wavelength photo-biomodulation in controlling post-surgical/post-treatment side-effects in dentistry.

In this respect, there would be two promising advantages in the usage of spectral technology: 1) the procedure of photobiostimulation is less time-consuming since the device makes it possible to treat larger surfaces and does not require the clinicians to manually positioning the optical fiber tip over the interested area; 2) it guarantees the established energy dose on the treated surface eliminating the intra/inter operator error, thus increasing the reproducibility of the photobiostimulation procedure.

The results of the present study would corroborate the usage of photobiostimulation, as a complementary therapeutic procedure, to alleviate pain and swelling after implant surgery. Further studies are needed to comparatively assess if spectral technology is as effective as conventional single wavelength photobiomodulation in controlling post-surgical/post-treatment side-effects in dentistry.
